# Potential mechanisms of formononetin against inflammation and oxidative stress: a review

**DOI:** 10.3389/fphar.2024.1368765

**Published:** 2024-05-10

**Authors:** Meiling Ding, Yiwen Bao, Huan Liang, Xiongwei Zhang, Bin Li, Ruocong Yang, Nan Zeng

**Affiliations:** ^1^ State Key Laboratory of Southwestern Chinese Medicine Resources, Chengdu, China; ^2^ Pharmacy College of Chengdu University of Traditional Chinese Medicine, Chengdu, China

**Keywords:** formononetin, oxidative stress, inflammation, mechanism, derivate

## Abstract

Formononetin (FMNT) is a secondary metabolite of flavonoids abundant in legumes and graminaceous plants such as *Astragalus mongholicus* Bunge [Fabaceae; Astragali radix] and *Avena sativa* L. [Poaceae]. Astragalus is traditionally used in Asia countries such as China, Korea and Mongolia to treat inflammatory diseases, immune disorders and cancers. In recent years, inflammation and oxidative stress have been found to be associated with many diseases. A large number of pharmacological studies have shown that FMNT, an important bioactive metabolite of Astragalus, has a profoundly anti-inflammatory and antioxidant potential. This review focuses on providing comprehensive and up-to-date findings on the efficacy of the molecular targets and mechanisms involve of FMNT and its derivatives against inflammation and oxidative stress in both *in vitro* and *in vivo*. Relevant literature on FMNT against inflammation and oxidative stress between 2013 and 2023 were analyzed. FMNT has antioxidant and anti-inflammatory potential and shows mild or no toxicity in various diseases. Moreover, in the medical field, FMNT has shown potential in the prevention and treatment of cancers, neurological diseases, fibrotic diseases, allergic diseases, metabolic diseases, cardiovascular diseases, gastrointestinal diseases and autoimmune diseases. Thus, it is expected to be utilized in more products in the medical, food and cosmetic industries in the future.

## 1 Introduction

Inflammation and oxidative stress have been implicated as key mechanisms in a range of disease processes. Several studies have presented the scientific basis about the interconnecting paths governing oxidative stress-induced inflammation and *vice versa*. Therefore, it has become imperative to selectively develop therapies and drugs for many diseases based on the relevant mechanisms (Khansari et al., 2009; Reuter et al., 2010; Salzano et al., 2014).

**FIGURE 1:**
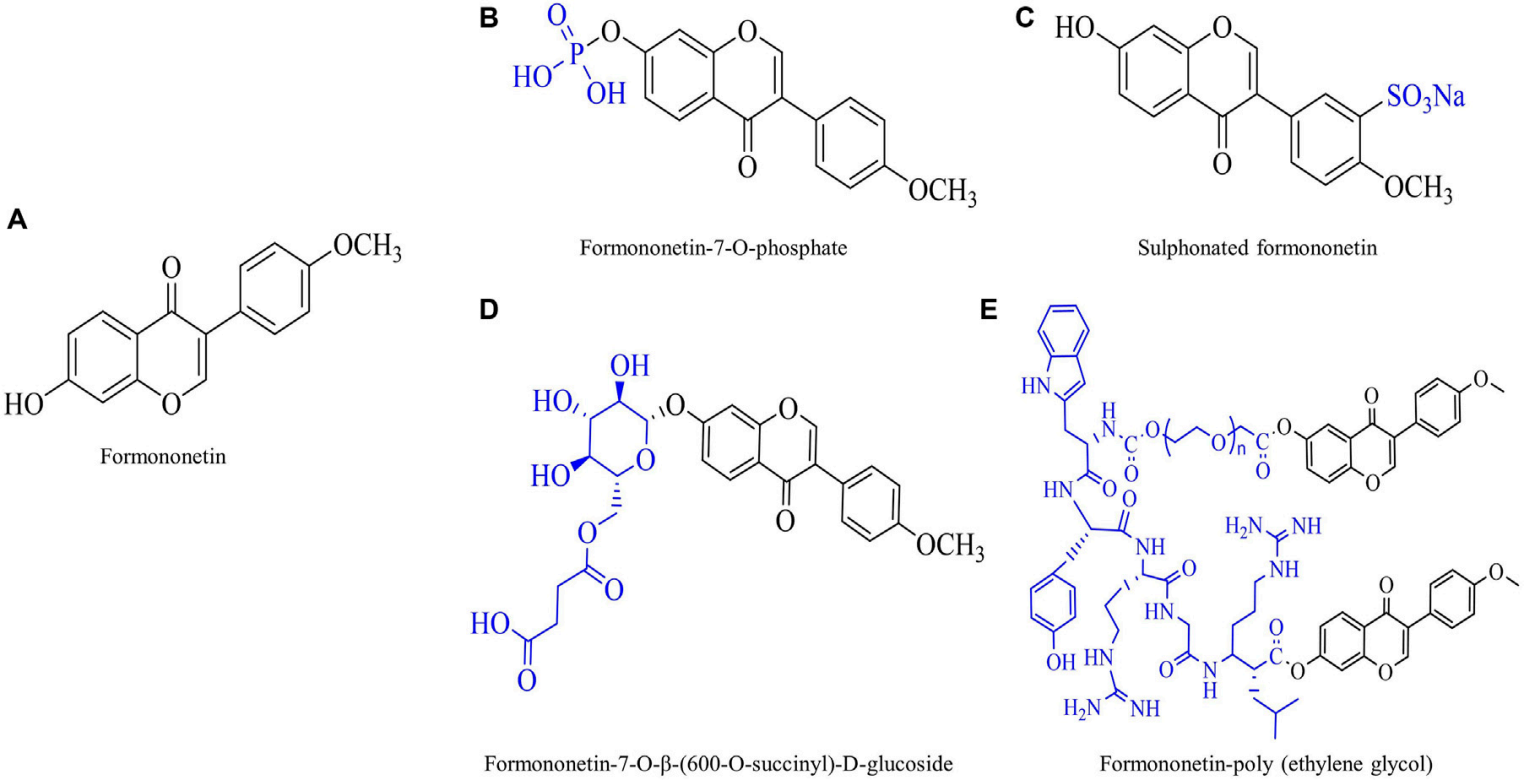
Chemical structure of formononetin (FMNT) and its derivatives. **(A)** Chemical structure of FMNT. **(B)** Chemical structure of FMNT-7-O-phosphate. **(C)** Chemical structure of sulphonated FMNT. **(D)** Chemical structure of FMNT-7-O-β-(600-O-succinyl)-D-glucoside. **(E)** Chemical structure of FMNT-poly (ethylene glycol).

Traditional botanical medicine is extensively used in the cure of inflammation and oxidative stress-related diseases due to its multi-metabolite, multi-target, multi-pathway and mild toxicity attributes. This is why the traditional application of botanical drugs and their active ingredients has been a top priority when researchers tap into modern medicine. Formononetin (FMNT) is an isoflavone, a type of flavonoid, and is one of the main bioactive metabolites of many botanical drugs such as *Trifolium pratense* L. [Fabaceae] and *Astragalus mongholicus* Bunge [Fabaceae; Astragali radix]. The chemical structure of FMNT is shown in Figure 1.

Various studies have previously shown that FMNT has a wealth of pharmacological effects, including antioxidant effects, anti-inflammatory activity, neuroprotective effects, anti-cancer effects, healing action, osteogenic action, anti-microbial and anti-viral activity, and treatment of diabetes and hypertension. Inspired by traditional uses, many researchers have explored the efficacy and molecular mechanisms of FMNT for inflammation and oxidative stress-induced diseases through the use of *in vivo* and *in vitro* models.

Although the pharmacological effects of FMNT have been summarized to some extent by previous authors, there is no review focusing on the anti-inflammatory and antioxidant pharmacological effects of FMNT, as well as its intrinsic in-depth molecular mechanisms. Thus, in present review we will provide an up-to-date and comprehensive overview of the anti-oxidative stress and anti-inflammatory activities of FMNT and assess its molecular mechanisms. It is desired to provide a detailed scientific basis and fundemental for future research directions and application development.

## 2 Anti-inflammatory molecular targets and mechanisms of FMNT

Inflammation, an early host immune response mediated by cytokines released from immune cells, is a critically underlying pathological process that usually accompanies the development of a wide range of diseases. Generally, the inflammatory response is a protection of cells exposed to various stimuli, whereas excessive inflammation can induce severe organismal damage and even tumor. When cells are stimulated by inflammation, mitogen-activated protein kinase (MAPK) first transmits inflammatory signals from the cell surface to the cell, promoting the activation of downstream kinases extracellular signal-regulated kinase 1/2 (ERK1/2) and c-Jun N-terminal kinase (JNK) as well as the expression of forkhead box O3 (FOXO3a) protein, which translocates to the nucleus and participates in the transcription of related inflammatory factors. In parallel, activated ERK1/2 can also promote inhibitor of kappa B kinase (IκBα) phosphorylation, accelerate IκBα ubiquitination degradation, and release the p50/p65 complex. The p50/p65 complex is transported to the nucleus to regulate transcription. Moreover, activated JNK can also promote signal transducer and activator of transcription 3 (STAT3) phosphorylation after entering the nucleus. Phosphorylated STAT3 acts in concert with the p50/p65 complex and FOXO3a to promote the expression of interleukin (IL), tumor necrosis factor-α (TNF-α), nitric oxide (NO), Prostaglandin E2 (PEG2) and other inflammatory factors. In addition to the MAPK dependency, the nuclear factor kappa B (NF-κB) and janus kinase/signal transducer and activator of transcription (JAK/STAT) pathways are also directly activated by TNF-α, granulocyte colony-stimulating factor (G-CSF), or IL-6, regulating the activity of downstream transcription factors and related enzymes. These highly expressed inflammatory factors further activate related inflammatory pathways, creating a vicious cycle. Noteworthy, FMNT has been shown to quench these processes and reduce the production of inflammatory factors, thereby alleviating inflammation.

### 2.1 Inflammatory mediators secretion regulation

#### 2.1.1 Cytokine release modulators

Cytokines are vital cellular signaling molecules and mediators of the immune system that monitor and regulate immune and inflammatory responses through complex networks. Unusual cytokine levels can contribute to cytokine storms and disease. The level of cytokines is therefore of great value in the diagnosis and therapy of diseases (Liu et al., 2021). A wealth of studies have shown remarkable anti-inflammatory activity of FMNT on luxuriant inflammatory models induced by manifold inflammagens *in vivo* and *in vitro* (Table 1). The primary macrophage-secreted pro-inflammatory cytokine, such as TNF-α and multiple ILs, chemokines, adhesion molecules and NOsynthesized by iNOS via the L-arginine pathway, are widely accepted to be compactly implicated in the development and amplification of inflammation.

**TABLE 1:** Cytokine and chemokine inhibitory properties of FMNT.

Dosage	Molding reagent (method)	Model	Intervention subject	Duration	Mechanisms	Reference
10, 30 mg/kg	Operation (Feeney’s weight-drop model)	Traumatic brain injury	Male Wistar rats	7 days	IL-10 expression activation	Li et al. (2018)
10 and 20 mg/kg	Operation (Feeney’s weight-drop model)	Traumatic brain injury	Male Wistar rats	5 days	TNF-α and IL-6 reduction	Li et al. (2014)
100 mg/kg	Ritonavir	Hepatotoxicity	Male SD rats	14 days	IL-1β, IL-6, and TNF-α reduction	Alauddin et al. (2018)
1.25 μg/mL; 1.25 μg/mL	IL-1β; Operation (anterior cruciate ligament transection model)	Osteoarthritis	Chondrocytes; male Sprague Dawley rats	24 h; 4 or 8 weeks	IL-6 and TNF-α suppression	Xiong et al. (2021)
30, 100, and 300 mg/kg	Lipopolysaccharide; Carrageenan	Inflammation	RAW264.7; female Swiss mice	24 h; 4 h	MPO, NOx, TNF-α, and IL-6 reduction	Bottamedi et al. (2021)
0, 4, 8 or 16 μg/mL	*Streptococcus* suis serotype 2	*Streptococcus* suis infection	J774	6 h	IL-1β, IL-6, and TNF-α reduction	Wang et al. (2020)
10 and 20 mg/kg	Lipopolysaccharide	Acute Lung Injury	Male BALB/c mice	1–3 h	IL-6, and TNF-α reduction	Ma et al. (2013)
10 or 100 mg/kg	Hyperoxia	Hyperoxic acute lung injury	C57BL/6 mice	72 h	IL-1β and IL-6 reduction	Chen et al. (2021)
10 µM	IL-13	Inflammation	JME/CF15	24 h	IL-1β, IL-6, TNF-α, GM-CSF and Eotaxin reduction	Huang et al. (2021)
5, 10, 25, 50, and 100 μM	IL-1β	Inflammation	Chondrocytes	24 h	Fractalkine, IL-1α, IL-1β, IL-6, IL-10, LIX, MCP-1, MIP-3α, TIMP-1, TNFα, and VEGF downregulation	Cho et al. (2019)

It has been reported that FMNT had a neuroprotective effect on brain injury by alleviating neuroinflammation. For example, the researchers found that levels of inflammatory cytokines were not only reduced by FMNT in the injured brain tissues. In addition, the team enriched the intervention effect of FMNT on inflammation in serum and brain tissue of the cortical neurons in follow-up studies. This also reflects the critical role of FMNT in brain disease inflammation. (Li et al., 2014; Li et al., 2018). In addition, FMNT was also able to alleviate side effects of drugs with multiple organ toxicity, and organ damage due to serious diseases. FMNT improved liver dysfunction in rat models of Ritonavir induced hepatotoxicity via inhibiting irregular IL-1β, IL-6, and TNF-α secretion (Alauddin et al., 2018). Besides, researchers demonstrated pro-inflammatory cytokine inhibitory activity of FMNT against acute lung injury induced by LPS. The authors observed a downregulation of increased IL-6 and TNF-α levels in dose-dependent manners after FMNT administration (Ma et al., 2013). Besides that, pre-treatment with FMNT distinctly attenuated the hyperoxia-induced increase of pro-inflammatory cytokine secretion (Chen et al., 2021). Similarly, other researchers found that FMNT administration mitigated the overexpression of TNF-α and IL-1β in cisplatin-induced acute kidney injury models on male Wistar rats (Hao et al., 2021).

In addition to anti-inflammatory cytokine release *in vivo*, FMNT also has the potential to diminish the overexpression of pro-inflammatory cytokines in inflammatory models *in vitro*. Recently, researchers evaluated the effect of FMNT in treating osteoarthritis. According to the authors, 1.25 μg/mL FMNT treatment visibly lowered IL-1β secretion in IL-1β stimulated chondrocytes (Xiong et al., 2021). Notably, the FMNT used in the experiments in both *in vivo* and *in vitro* in this article is the same concentration, which is inconsistent with the doses of the ordinary article and deserves more rigorous consideration. The potential of FMNT in remedying inflammation has been evaluated using the RAW264.7 macrophage model triggered by LPS. According to the results, FMNT showed reduction of the NOx level in the inflammatory macrophages, exhibiting favorable antioxidant activity (Bottamedi et al., 2021). Other authors stated that 32 μg/mL FMNT significantly reversed the strong inflammatory response caused by *streptococcus* suis serotype 2 infection in macrophage-like J774 cells (Wang et al., 2020).

#### 2.1.2 Chemokine release modulators

The chemokines (or chemotactic cytokines) are a large family of small, secreted proteins that signal via G protein-coupled heptahelical chemokine receptors on the cell surface (Hughes and Nibbs, 2018). They act primarily by stimulating cell migration and are the “primary regulators” of the movement and positioning of many inflammation-associated cells such as macrophages, lymphocytes, neutrophils and dendritic cells in the body. Chemokines therefore play a remarkable role in the development of inflammatory responses *in vivo* in terms of leukocyte recruitment and are involved in all destructive or protective inflammatory responses (Zlotnik and Yoshie, 2012). The studies reporting the inhibitory actions of FMNT on chemokines are discussed (Table 1).

Some researchers have reported the chemokines inhibitory effect of FMNT. In this study, the authors noted that FMNT treatment reduced eotaxin and granulocyte-macrophage colony-stimulating factor (GM-CSF) secretion in JME/CF15 cells after IL-13 stimulation in a dose-dependent manner (Huang et al., 2021). In another study, the chemokine inhibitory features of FMNT also have been displayed in rat chondrocytes induced by IL-1β. According to the authors, FMNT lowered the level of several chemokines, including fractalkine, macrophage inflammatory protein-3α, and monocyte chemoattractant protein-1, and showed brilliant osteoarthritis relief (Cho et al., 2019).

### 2.2 Mechanism intervention

#### 2.2.1 COX2/PGE2 pathway inhibition

PGE2 produced by mPGES-1 plays a crucial role in inflammation (Chang and Meuillet, 2011; Finetti et al., 2012). Isoform two of cyclooxygenase (COX2) converts arachidonic acid (AA) to PGH2, an unstable metabolite, which is then isomerized by mPGES-1 to inflammatory PGE2. Thus, COX2 inhibition has a specific value in the onset and duration of inflammatory diseases. Several studies reporting COX2 inhibitory properties of FMNT are summarized in this section (Table 2).

**TABLE 2:** COX2/PGE2 inhibitory properties of FMNT.

Dosage	Molding reagent (method)	Model	Intervention subject	Duration	Mechanisms	Reference
5, 10, 25, 50, and 100 μM	IL-1β	Inflammation	Chondrocytes	24 h	NO, COX2 and PGE2 suppression	Cho et al. (2019)
40 μM	Oxidized low-density lipoprotein	Endothelial dysfunction	HUVECs	24 h	COX2 and eNOS downregulation	Zhang et al. (2021)
4 mg/mL	Lipopolysaccharide	Inflammation	RAW264.7	48 h	NO, iNOS, COX2 and PGE2 downregulation	Kim et al. (2019)
2.5, 5 and 10 μM	Lipopolysaccharide	Neuroinflammation	BV2	24 h	iNOS and COX-2 downregulation	El-Bakoush and Olajide (2018)
10, 20 and 40 mg/kg	Methotrexate	Renal dysfunction	Male Wistar rats	10 days	iNOS and COX-2 downregulation	Aladaileh et al. (2019)

Studies have proven that chondrocyte dysfunction, such as type II collagen degradation and inflammation, is associated with joint disease like rheumatoid arthritis and osteoarthritis (Menu and Vince, 2011). Researchers reported anti-inflammatory role of FMNT via primary Rat Chondrocytes. According to the authors, 25 and 50 μM FMNT markedly downregulated the expression of PGE2 as well as COX2 in primary rat chondrocytes treated with 10 ng/mL IL-1β, in which the specific production of PGE2 was inhibited by 98% ± 3% and 77% ± 2.2%, respectively compared to control group. Results above suggested the counteracting role of FMNT on IL-1β-induced catabolic pro-inflammatory effects in primary rat chondrocytes and may counteract IL-1β-induced articular cartilage degeneration (Cho et al., 2019).

Scholars investigated the protective effect of FMNT on ox-LDL-induced endothelial dysfunction via network pharmacology and experimental validation. According to the authors, FMNT had an effect on anesthetizing ox-LDL-induced inflammation and apoptosis in HUVECs through downregulation of COX2, eNOS, and cleaved caspase-3 expression (Zhang et al., 2021). FMNT treatment also suppressed the level of PGE2 in LPS-treated RAW264.7 macrophage cells in a dose-dependent manner (Kim et al., 2019). What’s more valuable is that the authors of this study synthesized a novel bioinnovative product (formononetin 7-O-phosphate), which has lower cytotoxicity and stronger anti-inflammatory activity compared to FMNT. Moreover, other researchers reported the influence of FMNT on neuroinflammation in LPS-treated BV2 microglia. According to the authors, FMNT dramatically decreased the level of PGE2, iNOS and COX2, providing strong support for the development of new compounds with selective neuroprotective effects in the brain (El-Bakoush and Olajide, 2018). In addition, the COX2 inhibitory properties of FMNT against methotrexate-induced acute kidney injury in Wistar rats were evaluated. The authors verified that rats treated with FMNT had enhanced anti-inflammatory features, compared with positive controls due to its capacity to significantly downregulate COX2 production (Aladaileh et al., 2019). All in all, these results provide evidence that FMNT is capable of blocking the conversion of arachidonic acid to PGE2 by inhibiting COX2, thereby ameliorating the inflammatory response. Additionally, the inhibition of COX2 may be the result of restrain of one or more inflammatory intracellular and intercellular signaling cascades.

#### 2.2.2 NF-κB pathway inhibition

NF-κB is a key transcriptional regulator that controls the expression of many inflammatory and immune genes. It is inactivated in the cytoplasm by binding to I-κB. Once activated by stimuli such as viruses, oxidants and inflammatory cytokines, NF-κB dissociates from I-κB and binds to a specific promoter in the nucleus, thereby regulating gene expression and activate the transcription of TNF-α, IL-6, IL-1β and COX2. Excessive activation of NF-κB is associated with cancers, immune cell dysplasia, delayed cell growth as well as many chronic inflammatory diseases, including rheumatoid arthritis, asthma, AIDS, atherosclerosis and neurodegenerative diseases. Thus, in this portion, we review several studies that have reported the inhibitory properties of FMNT on NF-κB (Table 3).

**TABLE 3:** NF-κB pathway inhibitory properties of FMNT.

Dosage	Molding reagent (method)	Model	Intervention subject	Duration	Mechanisms	Reference
0.1, 1 and 10 µM	Anti-DNP IgE	Allergic inflammation	RBL-2H3 cells	16 h	NF-κB and IkKα phosphorylation reduction	Xu and An (2017)
10, 20 and 40 mg/kg	Ovalbumin	Allergic Asthma	Female SPF BALB/c mice	29 days	NF-κB and JNK inhibition	Yi et al. (2020)
25 mg/kg	Complete Freund’s adjuvant	Inflammatory pain	Male C57BL/6 mice	8–10 days	NF-κB signaling pathway inhibition	Wang et al. (2019)
50 mg/kg; 100 mg/kg	Concanavalin-A	Autoimmune hepatitis	Male BALB/c mice	10 days	NF-κB signaling pathway inhibition	Liu et al. (2021)
10, 30 and 60 mg/kg	Monocrotaline	Pulmonary arterial hypertension	Male Sprague-Dawley rats	2 weeks	Phosphorylated ERK and NF-κB decrease	Wu et al. (2020)
10, 20 and 30 mg/kg	Lipopolysaccharide	Mastitis	Female C57BL/6 mice	25 h	NF-κB downregulation	Xiang et al. (2022)

Allergic diseases, which include food allergies, asthma, anaphylaxis, sinusitis and atopic dermatitis, are triggered by allergens and feature adaptive allergic inflammation. In a study, the NF-κB inhibitory role of FMNT was assessed in a mast cell-mediated allergic inflammation model. Results indicated that the properties of FMNT were correlated with a restriction of NF-κB activation and upstream IκKα phosphorylation in a dose-dependent manner (Xu and An, 2017). However, this study was lack of the exploration of the dosage of FMNT, which was addressed in other articles. The NF-κB inhibitory effect of FMNT in an ovalbumin-induced asthmatic female BALB/c mice model was also been evaluated. According to the authors, FMNT treatments appeared to observably inhibit the activation of NF-κB, certifying distinct therapeutic effects of FMNT in attenuating airway inflammation (Yi et al., 2020). A20 is a negative regulator of NF-κB, which plays an integral role in the regulation of immune and inflammatory responses (Priem et al., 2020). In another study, researchers supplied evidence of the anti-inflammatory ability of FMNT against FITC-treated atopic dermatitis mice model as well as siA20 and siGPER-transfected HaCaT cells model. As reported, FMNT (10 mg/kg, i.p.) was capable of attenuating atopic dermatitis by enhancing A20 expression via GPER activation (Yuan et al., 2021). All of the above studies indicated that FMNT had a clear therapeutic effect on allergic inflammation.

A study investigated the anxiolytic role of FMNT against chronic inflammatory pain responses in C57BL/6 male mice. The FMNT administration has shown to inhibit NF-κB signaling and microglial activation in the mice with chronic inflammatory pain, thereby relieving the anxiety (Wang et al., 2019). Meanwhile, FMNT also has a non-negligible role in the regulation of autoimmune diseases. For example, studies showed that FMNT decreased the level of p-NF-κB p65 and increased the level of IκBα, the inhibitor of NF-κB, in the liver from concanavalin-A-induced autoimmune hepatitis mice, showing a powerful immunomodulatory property (Liu et al., 2021). FMNT also possess activity of restraining monocrotaline-treated pulmonary arterial hypertension, whose pharmacological mechanism was related to the downregulation of phosphorylated ERK and NF-κB (Wu et al., 2020). In addition to the inflammation model mentioned above, NF-κB pathway plays a critical role in mastitis. Scholars determined the protective effect of FMNT in LPS-induced mastitis in female lactating C57BL/6 mice and mouse mammary epithelial cells EpH4-Ev, and they observed an obvious inhibition of NF-κB signaling pathway activation in both *in vitro* and *in vivo* experiments (Xiang et al., 2022).

#### 2.2.3 MAPK pathway inhibition

The MAPK signaling pathway, a highly conserved serine/threonine protein kinase, is one of the most fundamental regulatory mechanisms in eukaryotic cells and part of a vital signaling system (Krishna and Narang, 2008). There are three well-characterized subfamilies of MAPKs in mammals: the ERKs, the JNKs and the p38 kinases (Cargnello and Roux, 2011). Of the three types of MAPKs, ERKs and JNKs are closely associated with some physiological processes, such as immunity, inflammation and tissue growth.

Several studies have shown the inhibition of FMNT to the activation of MAPK signaling pathways (Table 4). For instance, researchers revealed that FMNT administration was capable of down-regulating the expression of JNK1, ERK1 and p38α, which are compactly associated with MAPK pathway in LPS-induced inflammatory zebrafish models, thus showing a dramatic anti-inflammatory activity (Luo et al., 2019). Besides, in another experiment, the potential of FMNT for ameliorating cholestasis has been assessed. This study found that FMNT attenuated alpha-naphthyl isothiocyanate-induced inflammatory response through inactivation of JNK inflammatory pathway in a PPARα-dependent manner (Yang et al., 2019).

**TABLE 4:** MAPK, JAK-STAT and NLRP3 inflammasome inhibitory properties of FMNT.

Dosage	Molding reagent (method)	Model	Intervention subject	Duration	Mechanisms	Reference
0, 0.97, 1.95, 3.9, 7.8, 15.6 and 31.25 μg/mL	Lipopolysaccharide	Inflammation	Transgenic neutrophils fluorescent zebrafish	3 h	MyD88 or TRIF MAPK/ERK and MAPK/JNK downregulation	Luo et al. (2019)
10, 20 and 50 mg/kg	α-naphthyl isothiocyanate	Cholestasis	Male C57BL/6 J mice	10 days	JNK signaling pathway inhibition	Yang et al. (2019)
30 mg/kg	Operation (middle cerebral artery occlusion model)	Cerebral ischemia-reperfusion injury	Male SD rats	3 days	p-JAK2 and p-STAT3 suppression	Yu et al. (2022)
20 and 40 mg/kg	U266 cells	Multiple myeloma	Female athymic nu/nu mice	20 days	STAT3/5 abrogation	Kim et al. (2018)
10, 20 and 40 μM	Immunoglobulin E	Allergic inflammation	RBLs	1 h	NLRP3 Inflammasome inhibition	Wang et al. (2018)
25 and 50 μM	TNF-α	Acute colitis	HCT-116	12 h	NLRP3 Inflammasome inhibition	Wu et al. (2018)

#### 2.2.4 JAK-STAT pathway inhibition

JAK/STAT signaling pathway, one of the key central communication nodes for many cellular functions and processes, is a means of intracellular signaling in response to growth hormones and cytokines (Darnell, 1997). Its aberrant phosphorylation triggers the overexpression of key mediators associated with various autoimmune and inflammatory diseases (Liongue and Ward, 2013; Schwartz et al., 2017). Several of these studies presenting the JAK-STAT inhibitory effects of FMNT are reviewed in this section (Table 4).

In a study, researchers tested anti-inflammatory properties of FMNT on cerebral ischemia-reperfusion injury model in SD rats. Using Western blotting they found that FMNT inhibited the protein expression of p-JAK2 and p-STAT3 at a dose of 30 mg/kg body weight, indicating prominent JAK2/STAT3 signaling pathway inhabitation (Yu et al., 2022). In another study, FMNT was shown to lessen constitutive STAT3 and STAT5 activation in U266 and RPMI 8226 cells belonging to myeloma cell line after FMNT therapy. More interestingly, the raised production of reactive oxygen species (ROS) due to a GSH/GSSG imbalance was involved in above effect (Kim et al., 2018).

#### 2.2.5 NLRP3 inflammasome inhibition

Inflammasome is a group of intracellular polymeric protein complexes that activate inflammatory caspase-1. Its activation is a major inflammatory pathway and a key participant in innate immunity discovered in recent years (Franchi et al., 2009). Several inflammasomes have been identified, including NLRP2, NLRP7 and NLRP12, of which NLRP3 is the best characterized (Khare et al., 2012; Vladimer et al., 2012; Minkiewicz et al., 2013). NLRP3 is essential for host immune defense and is closely related to a variety of autoimmune and autoinflammatory diseases when it is disrupted (Menu and Vince, 2011). Several of these studies presenting the NLRP3 inflammasome inhibitory effects of FMNT are reviewed in this section (Table 4).

In a recent study, scholars explored therapies for lung injury and fibrosis and found that FMNT nanomedicines could block macrophage pyrodeath involved in NLRP3, thereby improving lung function and longevity in mice (Ouyang et al., 2023). FMNT was also found to suppress streptozotocin-induced NLRP3 protein overexpression in diabetic male C57BL/6 J mice (Wang et al., 2018). Other scholars assessed the NLRP3 inflammasome signaling pathway inhibitory activity of FMNT *in vitro*. They discovered that FMNT (25 μM, 50 μM) treatment to acute injury model of human colon carcinoma cell line (HCT-116) induced by TNF-α for 12 h led to the downregulation of NLRP3 inflammasome expression levels. Furthermore, they also performed simultaneous *in vivo* experiments and found that NLRP3 expression was diminished in the colon tissue of dextran sulfate sodium-induced acute colitis mice after FMNT treatment. This finding is consistent with the *in vitro* experiments (Wu et al., 2018).

**FIGURE 2:**
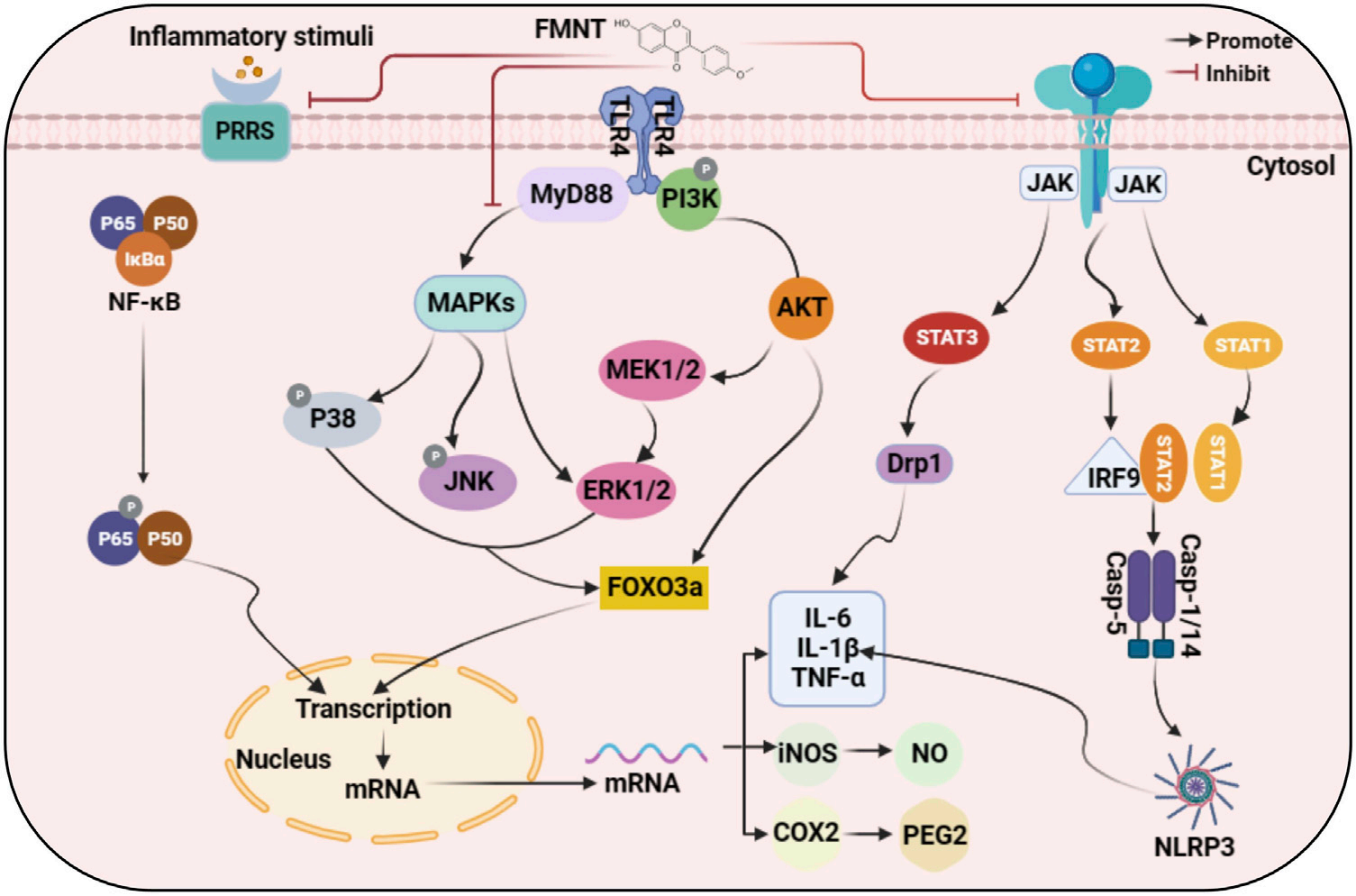
Molecular mechanism of anti-inflammatory action of FMNT.

The above evidence suggests that FMNT is a very promising anti-inflammatory agent that can inhibit the release of cytokines and chemokines by down-regulating the over-activated COX2/PGE2 pathway, NF-κB pathway, MAPK pathway, JAK-STAT pathway and reducing the production of NLRP3 inflammasome. It is well known that FMNT can be used in the treatment of multiple diseases such as liver injury, kidney injury, central nervous system and airway inflammation. Based on *in vitro* and *in vivo* studies of the mechanisms involved, we have summarized schematically the interaction pathways for the anti-inflammatory activity of FMNT as shown in Figure 2.

## 3 Anti-oxidative stress molecular targets and mechanisms of FMNT

Oxidative stress is an imbalance between oxidants and antioxidants that tends to favor oxidants, which leads to dysfunction of redox signaling and control and/or molecular damage (Sies et al., 2017). Oxidative stress can be triggered in a variety of diseases, including liver diseases, cardiovascular diseases and psychiatric disorders (Ng et al., 2008; Magenta et al., 2013; Jadeja et al., 2017). Noxious external stimuli first causes a large accumulation of ROS in the cell. On the one hand, excess ROS promotes the phosphorylation of its downstream AKT by activating phosphatidylinositol-3-kinase (PI3K). Normally, Kelch-like ECH-associated protein1 (Keap1) binds to nuclear factor erythroid-2 related factor 2 (Nrf2) in the cytoplasm in an inactive state. Phosphorylated AKT acts on the Keap1-Nrf2 conjugate, destabilizing its binding and releasing Nrf2. Free Nrf2 is translocated to the nucleus and binds to antioxidant response element (ARE), activating the transcription of downstream genes and increasing the expression of heme oxygenase-1 (HO-1) and antioxidant enzymes. On the other hand, excess ROS can also activate silent information regulator 1 (SIRT1). Activated SIRT1 directly promotes the deacetylation and subsequent activation of Nrf2, ultimately leading to the upregulation of Nrf2 target genes and increased antioxidant capacity. At the same time, excessive ROS can also increase the gene expression of peroxisome proliferator-activated receptor gamma (PPARγ), further enhancing antioxidant capacity. Interestingly, FMNT has been demonstrated to have the potential to promote these processes, leading to the production of more antioxidant enzymes and alleviating the damage to the body by oxidative stress.

### 3.1 ROS inactivation

ROS in the body refers to the general term of oxygen-containing and active substances composed of oxygen. Normal intracellular ROS acts as messengers in gene expression, signal transduction, and homeostasis. When ROS is excessive, oxidative stress will be triggered, which destroys many of the biological macromolecules in the cell and leads to apoptosis. High level of ROS leads to the formation of free radicals, which are highly oxidizing and can damage the body’s tissues and cells, leading to chronic disease and the effects of aging (Liochev, 2013).

FMNT may reduce oxidative stress in liver disease, showing hepatoprotective potential. Mitochondrial dysfunction produces excess ROS, while FMNT promoted PHB2/PINK1/Parkin-mediated mitophagy pathway, reduced the production of ROS and then alleviated ischemia/reperfusion-induced liver injury (Ma et al., 2022). The H_2_O_2_ molecule is a precursor of free radicals that can cause cell death (Halliwell, 1989). A study explored the inhibiting effect of FMNT on oxidative stress in H_2_O_2_-treated Chang Liver cells. The authors discovered that H_2_O_2_ decreased antioxidant activity and increased ROS levels, which was reversed after FMNT treatment (Li et al., 2015). The study lacks of reliability because it did not mention the dosage of FMNT. Ritonavir is a protease inhibitor of human immunodeficiency virus that has multiple adverse effects, including hepatotoxicity. Fortunately, FMNT was found to be succeed in mitigating ritonavir-induced hepatotoxicity through lowering the formation of ROS, thereby reducing its side effects and increasing its safety (Alauddin et al., 2018). In addition, researchers observed the gastric protective effect of FMNT (10 mg/kg) on a gastric ulcer rat model induced by ethanol and non-steroidal anti-inflammatory drugs. In this study, they were surprised to find that FMNT significantly reduced the total gastric injury area and gastric fluid secretion, and increased the amount of mucus secretion in rats with gastric ulcer by scavenging free radicals produced by oxidative stress (Mendonça et al., 2020). Besides, FMNT (40 mg/kg) also markedly alleviated airway oxidative stress in mice with allergic asthma by reducing ROS (Yi et al., 2020).

### 3.2 Enzymatic antioxidants activation

Enzymatic antioxidants, comprised of CAT, GSH-Px, SOD and Trx systems, provide a more efficient protection against brisk oxidative attacks due to their competence to break down ROS. By degrading the hydrogen peroxide into molecular oxygen and water, CAT has the capacity to neutralise the hydrogen peroxide. The main biomolecular function of GSH-Px is to remove lipid hydroperoxides, protecting cells from oxidative damage (Dokic et al., 2012). As for SOD, the most powerful antioxidant in the body, can be categorised into mitochondrial Mn-SOD, cytosolic CuZn-SOD and extracellular SOD (He et al., 2018). As a result, these antioxidases play immeasurable roles in pathological states that include I/R injury, viscera injury, acute myocardial infarction, traumatic brain injury and inflammation (Table 5).

**TABLE 5:** ROS inactivated and enzymatic antioxidants activated properties of FMNT.

Dosage	Molding reagent (method)	Model	Intervention subject	Duration	Mechanisms	Reference
1 mg/L	H_2_O_2_	Oxidative stress	Chang Liver	6 h	ROS inhibition	Li et al. (2015)
100 mg/kg	Ritonavir	Hepatotoxicity	Male SD rats	14 days	ROS and MDA inhibition; GSH activation	Alauddin et al. (2018)
10 mg/kg	Absolute ethanol; indomethacin	Gastric ulcer	Wistar rats of both sexes	30 min; 6 h	DPPH inhibition	Mendonça et al. (2020)
10, 20 and 40 mg/kg	Ovalbumin	Allergic Asthma	Female BALB/c mice	29 days	ROS inhibition; SOD activation	Yi et al. (2020)
25 and 50 mg/kg	Gene Screening	Hepatotoxicity	Male db/db mice	8 weeks	MDA decrease; SOD, GSH-Px, and CAT activation	Lv et al. (2020)
20 mg/kg	Isoproterenol	Acute ischemic injury	BALB/c mice of both sexes	4 weeks	LDH decrease; SOD and CAT activation	Zhao et al. (2021)
5, 10 and 20 mg⁄kg	Operation (left coronary artery ligation model)	Acute myocardial infarction	Male SD rats	6 h	SOD and GSH-Px activation	Zhang et al. (2011)
-	H_2_O_2_	Apoptosis	RGC-5	24 h	8-OHdG, ROS and MDA activation; MnSOD activation	Jia et al. (2014)

Study found that FMNT could stimulate the activation of the Nrf2 pathway, producing the majority of cytoprotective proteins. These processes regulate the transcription of antioxidant genes (such as SOD and GSH-Px) through a series of biochemical cascades, thereby alleviating neuroinflammation and playing a neuroprotective role (Li et al., 2014). Another study revealed that FMNT was able to elevate levels of SOD, CAT and GSH-Px in db/db mice, alleviating diabetic nephropathy (Lv et al., 2020). Studies of ischemia reperfusion showed apparent roles of FMNT in improving the activities of CAT, SOD and GSH-Px, and relieved damage of the cell membrane and increase in permeability evoked by superfluous oxygen-free radicals (Zhao et al., 2021; Ma et al., 2022). Except for ischemia-reperfusion, oxidative stress caused by overproduction of ROS and depletion of antioxidants in the defense system has also been implicated in the pathogenesis of acute myocardial infarction (AMI). Not surprisingly, FMNT reduced H_2_O_2_-induced apoptosis, via increasing the level or activity of antioxidant enzymes and inhibiting the activation of NF-κB, a key transcription factor for apoptosis (Jia et al., 2014). In addition, sodium formononetin-3′-sulphonate (Sul-F), a derivative of FMNT, has been reported to boost the expression of antioxidant enzymes that were consumed in heart tissue in the AMI model (Zhang et al., 2011). However, in this article, the author did not make comparison with FMNT and could not directly observe the advantages of Sul-F. Taken together, these findings suggested that FMNT may serve as a promising drug candidate to exert antioxidant effects in a variety of acute and chronic diseases.

### 3.3 Nrf2/HO-1 pathway activation

Nrf2 is an emerging cellular antioxidant regulator, controlling the basal and inducible expression of a number of antioxidant response element-dependent genes, regulating the physiological and pathophysiological consequences of ROS exposure (Ma, 2013). HO-1 is a heme degradation enzyme, and heme metabolites can act as antioxidative mediators of HO-1 cell protection, reducing ROS damage. As an indispensable regulatory pathway for intracellular antioxidative stress defense, Nrf2/HO-1 signaling pathway is actively engaged in apoptosis, anti-inflammation, antioxidation and other processes (Table 6), making it of great interest (Morita, 2005; Luo et al., 2019).

**TABLE 6:** Nrf2/HO-1 pathway activated properties of FMNT.

Dosage	Molding reagent (method)	Model	Intervention subject	Duration	Mechanisms	Reference
10 and 30 mg/kg	Operation (Feeney’s weight-drop model)	Traumatic brain injury	Male Wistar rats	5 days	Nrf2-dependent antioxidant pathways activation	Li et al. (2017)
20, 40 μM; 50 and 100 mg/kg	Acetaminophen; acetaminophen		LO2; Male BALB/c mice	30 h; 7 days	Nrf2 activity enhancement	Jin et al. (2017)
10 or 100 mg/kg	Hyperoxia	Hyperoxic acute lung injury	C57BL/6 mice	72 h	Nrf2 activation	Chen et al. (2021)
10, 20 and 40 mg/kg	Methotrexate	Renal dysfunction	Male Wistar rats	10 days	Nrf2/HO-1 Upregulation	Aladaileh et al. (2019)
60 mg/kg	Gentamicin	Nephrotoxicity	Male Wistar rats	2 weeks	Nrf2 upregulation	Althunibat et al. (2022)
75 mg/kg	Cisplatin	Acute kidney injury	Male Wistar rats	5 days	PPARα/Nrf2/HO-1/NQO1 pathway activation	Hao et al. (2021)

Oxidative stress is considered a major contributor to traumatic brain injury. A study assessed the HO-1 stimulatory properties of FMNT in rats with traumatic brain injury and found that FMNT pretreatment for 5 days can significantly upregulate HO-1 expression in model rats, showing neuroprotective property (Li et al., 2017). In addition, another study reported the Nrf2 stimulatory activities of FMNT in human non-tumor hepatic cells LO2 and an acetaminophen-induced hepatotoxicity mouse model. The authors observed that FMNT had the potential to enhance Nrf2 expression in both *in vivo* and *in vitro* liver disease models (Jin et al., 2017). In parallel, FMNT treatment was able to enhance Nrf2 level and then upregulate HO-1 expression in hyperoxic acute lung injury mice. To verify this result, Nrf2 inhibitors was administered to pulmonary microvascular endothelial cells and found that when Nrf2 was silenced, the upregulation of HO-1 by FMNT was abrogated (Chen et al., 2021). Several studies have revealed that FMNT treatment had the ability to upregulate Nrf2/HO-1 signaling and prevented oxidative stress in rats with kidney damage caused by different factors, exhibiting renal protection (Aladaileh et al., 2019; Hao et al., 2021; Althunibat et al., 2022).

### 3.4 PI3K/AKT pathway regulation

Elevated levels of ROS induced oxidative stress can lead to cell death. Akt is a serine/threonine protein kinase phosphorylated and activated by extracellular factors in a PI3K-dependent way (Yang et al., 2017). As a key inhibitor of apoptosis, the PI3K/Akt signaling pathway has been shown to play an indispensable role in participating in cell metabolism and promoting cell survival (Table 7), thereby alleviating oxidative stress (Garcia-Echeverria and Sellers, 2008).

**TABLE 7:** PI3K/AKT, SIRT1 and PPARγ activated properties of FMNT.

Dosage	Molding reagent (method)	Model	Intervention subject	Duration	Mechanisms	Reference
25, 50 mg/kg	Operation (flap model)	Flap necrosis	Male C57BL/6 mice	7 days	PI3K/Akt activation	Li et al. (2022)
50 mg/kg; 100 mg/kg	Concanavalin-A	Autoimmune hepatitis	Male BALB/c mice	10 days	PI3K/AKT activation	Liu et al. (2021)
0, 10, 20, and 40 μM	Bromodeoxyuridine	Ovarian cancer cell proliferation	ES2 and OV90	48 h	PI3K/AKT activation	Park et al. (2018)
25 and 50 mg/kg; 0, 5, 10, 20, 40, 60, 80, 100 and 150 μM	Gene Screening; Hyper-glycemic conditions	Diabetic renal fibrosis	Male db/db mice; GMCs	8 weeks; 24 h	SIRT1 upregulation	Zhuang et al. (2020)
10, 20 and 40 mg/kg	high fat diet + low dose of streptozotocin	Diabetic nephropathy	Male SD rats	16 weeks	SIRT1 upregulation	Oza and Kulkarni (2019)
30 µM	Lipopolysaccharide	Release of high mobility group box 1	RAW264.7	24 h	SIRT1 upregulation	Hwang et al. (2018)
10 and 20 mg/kg	Lipopolysaccharide	Acute Lung Injury	Male BALB/c mice	1–3 h	PPARγ Induction	Ma et al. (2013)
40 μM	Oxidized low-density lipoprotein	Endothelial dysfunction	HUVECs	24 h	PPAR-γ signaling activation	Zhang et al. (2021)

Flap necrosis is one of the main post-operative complications. In a recent study, FMNT (25 and 50 mg/kg) was inhibition of oxidative damage and enhancement of the survival of random skin flaps by activating PI3K/Akt-driven Nrf2 antioxidant defense system in a dose-dependent manner (Li et al., 2022). What’s more, researchers reported that FMNT could ameliorate chronic kidney disease-induced muscle atrophy and TNF-α-induced C2C12 myotubes via reversing myostatin-generated dephosphorylation of the PI3K/Akt pathway as well as the defective proliferation and differentiation behavior of satellite cells (Liu et al., 2021). FMNT is also known to induce cancer cells to apoptosis. FMNT was found to be able to inactivate PI3K/AKT pathway and restrained ovarian cancer cell proliferation through arrest in the sub-G0/G1 cell phase. Moreover, FMNT induced cell apoptosis involving DNA fragmentation through MMP degradation and ROS accumulation, with subsequent P38 activation in OV90 and ES2 cells (Park et al., 2018).

### 3.5 SIRT1 activation

As a class III nicotinamide adenine dinucleotide (NAD)-dependent histone deacetylase, SIRT1 has been shown to regulate critical metabolic activities such as apoptosis, ageing and oxidative stress by deacetylating a diverse range of zymolytes (Oberdoerffer et al., 2008; Yu and Auwerx, 2010). An elevation of ROS activates SIRT1, which in turn leads to a decrease of the ROS (Zhang et al., 2017). Therefore, SIRT1 plays a considerable role in anti-oxidative stress, which has been validated by quite a few studies (Table 7).

Type 2 diabetes brings multiple complications in the clinic, the most common of which are vascular complications, including diabetic nephropathy, diabetic neuropathy, and more, most of which are affiliated with inactivated SIRT1. In a study, Male db/db mice were gavaged with 25 and 50 mg/kg/day FMNT for 8 weeks and sacrificed. While the result emerged that FMNT upregulated the level of quenched Sirt1 to activation of the Nrf2/ARE pathway (Zhuang et al., 2020). In other studies, Oza assessed the SIRT1 stimulatory properties of FMNT to kidney damage and neuropathy in type 2 diabetes rats, and spotted that the low expression of SIRT1 was observably reversed after administration with FMNT for 16 weeks (Oza and Kulkarni, 2019; 2020). Above articles illustrated an efficacious antioxidation of FMNT in the control of type 2 diabetes. Other researchers used an in-depth method of small interfering RNA-mediated gene silencing to explore the inhibition effect of FMNT in LPS-induced high-mobility group box 1 release through PPARδ-dependent activation of SIRT1 (Hwang et al., 2018). These findings have profound connotation for our comprehension of the molecular mechanism underlying the transcriptional regulation of SIRT1.

### 3.6 PPARγ activation

PPARγ is a member of the nuclear hormone receptor transcription factor superfamily and is an active participant in the regulation of genes involved in metabolism, inflammation, immunity, cell proliferation and differentiation (Willson et al., 2000; Schupp et al., 2009; Rogue et al., 2010). Oxidative stress induces a decline in PPARγ levels and activity by downregulating PPARγ transcription, potentially involved in the activation of inhibitory redox-regulated transcription factors (Blanquicett et al., 2010). Thus, elevation of PPARγ represents a promising strategy.

Previous studies have demonstrated that FMNT exerted a promoting effect on PPARγ (Table 7). In the case of acute lung injury mice induced by LPS, the downregulated PPAR-γ gene expression was obviously improved upon FMNT treatment (Ma et al., 2013). In addition, treatment with FMNT also resulted in a provoke of PPAR-γ signaling in ox-LDL-stimulated endothelial injury in HUVECs, thus supplying a theoretical basis for FMNT as a potential anti-atherosclerotic drug (Zhang et al., 2021). However, the results of this experiment were relatively simple, as only one concentration of FMNT (40 µM) was tested and no positive drugs were compared.

**FIGURE 3:**
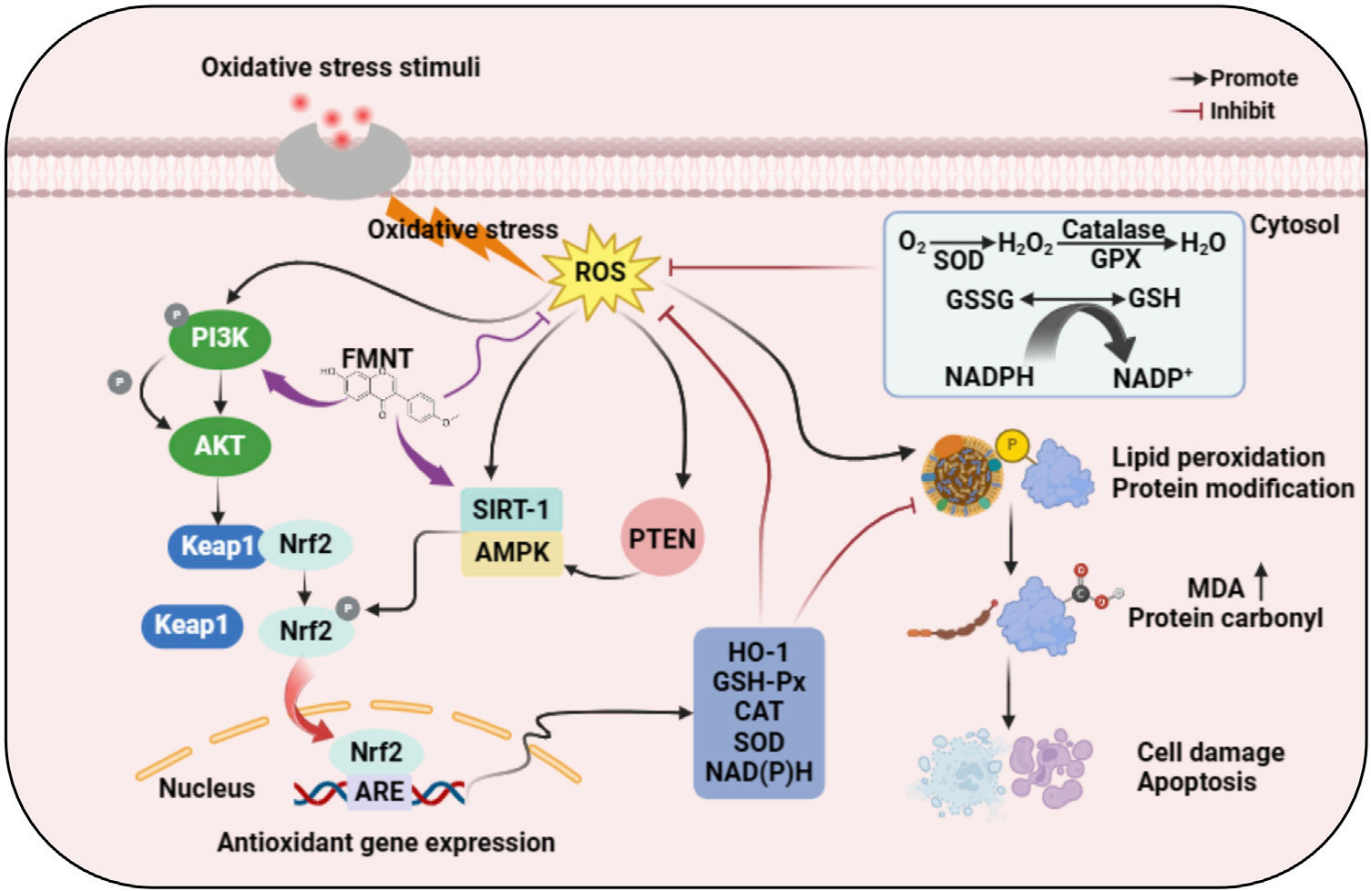
Molecular mechanism of anti-oxidative stress action of FMNT.

The above evidence implies that FMNT is a highly potent antioxidant that can stimulate the expression of antioxidant enzymes by promoting the activation of Nrf2/HO-1 pathway, PI3K/AKT pathway, SIRT1 and PPARγ, which in turn eliminates the excessive accumulation of ROS, the “killer” for oxidative stress, and alleviate ROS-induced tissue and cell damage. It plays an exceptional antioxidant role in various pathological models such as cancer, neurological diseases, fibrotic diseases, allergic diseases, metabolic diseases, cardiovascular diseases, gastrointestinal diseases and autoimmune diseases. We have summarized the interaction pathway of FMNT’s antioxidant activity based on previous studies of relevant mechanisms *in vitro* and *in vivo*, as shown in Figure 3.

## 4 Absorption, bioavailability, metabolism and toxicity of FMNT

The pharmacokinetic study of FMNT in rats showed that FMNT had poor oral activity, with a bioavailability of 21.8% after a single oral dose. FMNT is rapidly absorbed in the intestinal tract of animals, distributed throughout the body for a short period of time, then rapidly eliminated and metabolized to sulfate and glucuronide for excretion (Jeong et al., 2005; Luo et al., 2018). The relatively low bioavailability of FMNT may be due to its poor water solubility as well as its short half-life (2.1 h).

To date, the vast majority of reports have indicated that FMNT is non-toxic. However, when used with ultra-high doses, FMNT was found to cause low toxicity in some models. Researchers conducted a 90-day subchronic toxicity study using Sul-Fat doses of 0, 33.3, 100 and 300 mg/kg. The results of this study showed that intravenous administration of Sul-F at a dose of 300 mg/kg produced a white-crystal (non-metabolic Sul-F) and transient vomiting in Beagle dogs. Under these conditions, the no observed adverse effect level (NOAEL) of Sul-F was 100 mg/kg in dogs (Li et al., 2016). Recently an acute and sub-acute toxicity research of FMNT has been conducted in Swiss albino mice. During the acute toxicity trial, it was found that the LD_50_ for FMNT was assessed to be 103.6 mg/kg, with a NOAEL of 50 mg/kg, whereas mortality was existing in the 300 mg/kg dose group and pathological changes like a mild diffuse granular degeneration in the liver were noted. Noteworthily, during subacute study, there were no changes in the mice’s organ tissue structures or metabolism after 28 days of intraperitoneal injections of FMNT at doses of 12.5, 25 and 50 mg/kg (Pingale and Gupta, 2023).

## 5 Conclusion and future prospects

Traditional knowledge-based botanicals and their active metabolites are usually treated as one of the alternatives of therapy. In recent years, bioactive metabolites derived from natural plants have appealed great interest to researchers due to their wide range of anti-inflammatory and antioxidant properties. FMNT is a widely available isoflavone with a wide range of biological activities. It is currently in use and has several patent outputs in human and animal nutritional supplementation, crop fungicide, and weight control. Oxidative stress and inflammation are inextricably linked, so limiting oxidative stress is a strategy for controlling inflammation. This present study comprehensively reviews and summarizes the antioxidant and anti-inflammatory activities of FMNT and their relevant mechanisms of action *in vitro* and *in vivo*. It was found that FMNT has an excellent alleviating effect in inflammation accompanied with cancer, neurological diseases, fibrotic diseases, allergic diseases, metabolic diseases, cardiovascular diseases, gastrointestinal diseases and autoimmune diseases. However, the results of these effects are inconsistent and the doses used vary widely across reports, thus the results need to be critically assessed and analyzed as they depend on the level and type of health of the subject. Studies have shown that most human studies of isoflavones and flavonoids have been unsuccessful, mainly due to their poor oral bioavailability. Noteworthy, the researchers synthesized several derivatives of FMNT through chemical and biological modifications: FMNT-7-o-phosphate, sulphonated FMNT, FMNT-7-o-β -(600-O-succinyl) -d-Glucoside, and FMNT-poly (ethylene glycol). Their chemical structures are shown in Figure 1. These novel FMNT derivatives improve FMNT’s relatively low bioavailability, poor water solubility, and systemic toxicity, thereby enhancing its anti-inflammatory and antioxidant activity. More interestingly, coupling with targeting peptide, FMNT derivatives enable targeted and precise drug delivery. Taken together, we clearly understand that FMNT derivatives are more potent than FMNT, and in some of the literature we have found the benefits of long-term FMNT supplementation. Despite the strong anti-inflammatory and antioxidant potential of FMNT in disease models, more research is required into the side effects of long-term and heavy dose of FMNT in healthy tissues and cells before it can be considered for use in areas other than medicine.

At present, the mechanism research of FMNT still has the following shortcomings. First, there is a great variation in dosage, so it is necessary to explore the minimum active concentration of FMNT and standardize the dosage. Second, these articles are very original, but future research needs improvement in the normativity of species names. This is what most articles about FMNT lack of. Third, the signal pathways are relatively common, and the effects of FMNT on new or rare pathways are rarely explored. Finally, gene knockout and inhibitor research methods are also rarely used in published studies. To fully understand the mechanistic pathways behind the anti-inflammatory and antioxidant properties of FMNT, more research gaps need to be filled. Future perspectives should focus on investigating the optimal benefits of FMNT, especially recommendations for long-term intake and forms of intake. Its mild or no toxicity allows it to be formed into more products for commercial applications. For example, in the field of pharmaceuticals, it may be a lead molecule for the treatment of diseases related to oxidative stress and inflammation. Alternatively, as a material or supplement for the manufacture of anti-tumour, lipid-lowering, anti-allergy and immunosuppressive drugs. It can also be used to promote wound healing, reduce scar hyperplasia and infection and other post-operative sequelae by promoting skin flap survival. In the field of food, it may be used as dietary supplements for purposes of improvement of function, balance of metabolism and reduction of disease risk. As to the field of cosmetics, it is a wise choice to develop FMNT into cosmeceuticals related to the alleviation of inflammation and removal of acne in the skin. Consider the oxidation resistance of FMNT, it may be a wonderful innovation as a source for cosmeceuticals against skin aging. Research of FMNT in the future should focus on how to maximize the bioactive effects of FMNT with appropriate dosing regimens and supplementation forms for humans.

## Figures and Tables

**Table:** 

**FMNT**	formononetin
**PGE2**	Prostaglandin E2
**eNOS**	endothelial nitric oxide synthase
**TNF-α**	tumor necrosis factor-α
**IL-1β**	Interleukin—1β
**MAPK**	Microtubule-Associated Protein Kinase
**JNKs**	c-Jun N-terminal kinases
**LPS**	lipopolysaccharide
**GM-CSF**	granulocyte-macrophage colony-stimulating factor
**GSH**	glutathione
**NLRP3**	NACHT, LRR and PYD domains-containing protein
**Nrf2**	The nuclear factor erythroid-2 related factor 2
**Akt**	Protein kinase B
**DNA**	Deoxyribonucleic acid
**NAD**	nicotinamide adenine dinucleotide
**H**_**2**_ **O**_**2**_	Hydrogen peroxide
**SOD**	superoxide dismutase
**COX2**	Cyclooxygenase-2
**NF-κB**	Nuclear factor kappa B
**iNOS**	Inducible Nitric Oxide Synthase
**IL-6**	Interleukin-6
**GPER**	G protein-coupled estrogen receptor
**ERKs**	extracellular signal-regulated kinases
**NO**	nitric oxide
**ROS**	reactive oxygen species
**STAT**	signal transducer and activator of transcription
**JAK**	The Janus kinase
**PPAR**	peroxisome proliferator activated receptor
**HO-1**	The heme oxygenase-1
**PI3K**	phosphatidylinositol 3-kinase
**MMP**	Mitochondrial membrane potential
**SIRT1**	Recombinant Sirtuin 1
**CAT**	catalase transaminase
**LD**_**50**_	Lethal Dose 50
